# Attention Deficit/Hyperactivity Disorder in Adults with Sleep Apnea

**DOI:** 10.1007/s10880-012-9331-2

**Published:** 2012-11-24

**Authors:** Ömer Oğuztürk, Mehmet Ekici, Dilay Çimen, Aydanur Ekici, Erol Senturk

**Affiliations:** 1 Department of Psychiatry Diseases, Kirikkale University Faculty of Medicine, Kirikkale, Turkey; 2 Department of Pulmonary Diseases, Kirikkale University Faculty of Medicine, Ziya Gökalp Cad. Fabrikalar Mah. Umut Sitesi D Blok. Daire: 1, 07100 Kirikkale, Turkey

**Keywords:** OSA, Attention deficit/hyperactivity disorder, Quality of life, Anxiety, Depression

## Abstract

AAttention deficit hyperactivity disorder (ADHD) is a common childhood illness. In some patients, this illness may persist into adulthood and an association between ADHD and Obstructive Sleep Apnea (OSA) has been found in childhood. However, it is unclear how OSA and ADHD coincide in adulthood. Therefore, to explore the relationship between OSA and adult ADHD the current investigation utilized a clinically-based cross-sectional survey. Subjects consisted of 81 treatment-naïve OSA patients and 32 controls. Measures included each patient completed a questionnaire regarding sleep, Adult ADHD scale. Clinical information, body mass index, 36-item Short Form Health Survey (SF-36), Epworth Sleepiness Scale (ESS), Hospital Anxiety and Depression Scale, and polysomnography.The subjects with Apnea-Hypopnea Index (AHI) ≥ 5 events/h were defined as patients with OSA. The control group was accepted as individuals with AHI > 0 events/h. The prevalence of adult ADHD was not different between the patients with OSA and the control group [(7.4 % (6/75) vs. 6.3 % (2/30), *p* = 0.8, respectively]. OSA patients with ADHD, as compared with those without, had higher anxiety scores and poorer physical component scores of quality of life and higher ESS scores. ADHD scores in patients with OSA were associated with anxiety and depression scores and SF36 physical and mental component scores in bivariate analyses. Thus, in our sample ADHD was not a frequent illness in adult patients with OSA. However, in patients with OSA and ADHD higher levels of anxiety and daytime sleepiness and poorer quality of life was found.

## Introduction

Attention deficit hyperactivity disorder (ADHD) is a common childhood illness with prevalence between 3 and 16 % (Naseem, Chaudhary, & Collop, 2001). It is characterized by hyperactivity, impulsiveness, impairment in academic, social, and occupational functioning, short attention span, and onset of symptoms before age 7 years (Dopheide & Pliszka, 2009). In some patients, this illness can persist into adulthood (Wender, Wolf, & Wasserstein, 2001). Obstructive sleep apnea (OSA) is a common disorder affecting 3 % of the population and is characterized by hyper somnolence, snoring, disturbed sleep, and cognitive dysfunction (Riha, Gislasson, & Diefenbach, 2009; Mazza et al., 2005). Some studies have suggested an association between OSA and ADHD in children (Philipsen, Hornyak, & Riemann, 2006; Surman, Thomas, Aleardi, Pagano, & Biederman, 2006), however, due to the overlap of symptoms, patients with OSA may be incorrectly considered to have ADHD (Surman et al., 2006).

Daytime tiredness or sleepiness and deficits in cognitive performance are common complaints in sleep disordered patients (Alchanatis et al., 2005; Lojander, Kajaste, Maasilta, & Partinen, 1999; Mazza et al., 2005; Quan et al., 2006; Schneider, Fulda, & Schulz, 2004). Since primary sleep disorders are associated with cognitive impairment, one might expect that ADHD symptomatology may improve if comorbid sleep disorders are adequately treated in addition to specific ADHD treatment (Schredl, Alm, & Sobanski, 2007).

As ADHD often persists into adulthood and simultaneously ADHD and OSA may co-occur in childhood the current study investigated the prevalence of ADHD in adults with OSA.

## Method

### Subjects

113 subjects admitted to the Sleep Laboratory at the University Department of Medicine, Hospital, for overnight sleep studies, secondary to clinical referral for suspected sleep apnea, were recruited. Exclusion criteria included known diagnosis of diabetes mellitus on medications, acromegaly, chronic renal failure, systemic steroid treatment and hormonal replacement therapy, a history of bipolar disorder, psychosis, anxiety disorder, seizure disorder, substance abuse history or mental retardation. A questionnaire on demographics, sleep symptoms, medical history, and medications was completed. Body mass index (BMI) (kg/m^2^) was calculated by measuring weight and height. All subjects gave written informed consent. The study was approved by the local Ethics Committee. None of the patients or the controls had used nasal CPAP–BPAP due to OSAS.

### Polysomnography

Standard nocturnal polysomnography was performed with recording of sleep stages (electroencephalography, chin muscles electromyography, electro-oculography), measurements of oronasal airflow with nasal canule, snoring, respiratory movements and oxygen saturation (SaO_2_) with a finger pulse oximeter (Sleep Screen Pro, 27 channels). Sleep stages were manually scored using standard Rechtschaffen and Kales (1968) scoring guidelines. Apnea was defined as cessation of airflow for 10 s. Hypopnea was defined as a 30 % reduction of airflow or respiratory movements accompanied by a 3 % decrease in SaO_2_ and/or followed by an arousal. The threshold of 10 apnea and hypopnea per hour of sleep was chosen to define OSA. Patients with OSA were defined as those subjects with an Apnea-Hypopnea Index (AHI) > 10 events/h and the control group consisted of subjects with an AHI < 5 events/h.

### Adult Attention Deficit/Hyperactivity Disorder Scale (ADHD Scale)

The ADHD Scale consists of 2 subscales, inattention and hyperactivity impulsivity, each of which contains 9 items. The ADHD scale is based on the criteria for ADHD from the DSM-IV. The ADHD scale is an expanded rating scale of 0 to 3: 0 = never, 1 = sometimes, 2 = often, 3 = very often. Each item asks how often a symptom occurred over the past 6 months on a 4-point Likert scale: 0 for never, 1 for sometimes, 2 for often, and 3 for very often (Turgay, 1995). The validity and reliability of Turkish version of the ADHD scale has been performed by Günay et al. (2006).

Individuals with a sum score on the ADHD scale of 36 or greater are considered highly likely to have ADHD.

A cut off score of 36 or higher has been shown to identify 82.5 % of adults with ADHD (sensitivity), 90.8 % of the healthy controls (specificity) (Öncü, Ölmez, & Şentürk, 2005).

### Assessment of Psychological Distress

The participants were asked to fill in the self-reported Hospital Anxiety and Depression Scale (HADS) questionnaire for the assessment of psychological distress. The questionnaire consisted of 14 questions in which the overall severity of anxiety and depression was rated on a four-point scale (0–3). Seven questions were related to anxiety and seven to depression (Zigmond & Snaith, 1983).

The seven-item depression subscale score ranges between 0–21, 0–7 as a cut-off point = normal, 8–10 = mild, 11–14 = moderate, 15–21 = severe, indicating the possibility of a mood disorder.

The reliability and validity of the Turkish version of HAD was made by Aydemir, Guvenir, Kuey, and Kultur (1997).

In the first phase of their study the Scale was translated and back translated by bilingual experts. The following phase was the evaluation of the validity and reliability of HAD Scale in university students. In the last phase validity and reliability were tested in a group of 136 inpatients admitted to the internal medicine clinics. Testing the reliability of HAD scale. Cronbach alfa coefficient for anxiety subscale was 0.8525 and for depression subscale it was 0.77. Item total score correlation coefficients were ranging between 0.81 and 0.85 in anxiety subscale and 0.73 and 0.77 in depression subscale. The correlation coefficient of split-half was 0.85 for anxiety subscale and 0.80 for depression subscale. Testing the concurrent validity of anxiety subscale with Spielberger’s Trait Anxiety Inventory, correlation coefficient was 0.75 and of depression subscale with Beck Depression Inventory, it was 0.72. They performed factor analysis for construct validity and obtained to factors. First factor contained anxiety symptoms; and second factor, depression symptoms. Using ROC analysis, 7 was found to be the cut-off score for depression subscale and 10 for anxiety subscale. As a result of the study, Turkish version of HAD Scale is valid and reliable in medically ill patients.

### Epworth Sleepiness Scale (ESS)

The Epworth Sleepiness Scale (ESS) is a validated 8-item measure of daytime sleepiness. On the ESS respondents estimate how likely they are to doze in eight different situations. The scores are based on a 0-to-24-point scale, with higher scores representing greater levels of sleepiness. The ESS can discriminate the sleepiness level of OSA patients from normal (Johns, 1991).

The reliability and validity of the Turkish version of the ESS was made by Ağargün et al. (1999).

Forty patients with primary hypersomnia and 41 healthy control subjects were included in their study. Internal homogeneity of separate items was assessed using Cronbach’s a statistic and Pearson correlation analysis. Test-retest reliability was assessed with paired *t* tests and Pearson correlation analysis. Validity was assessed using Student’s *t* test. The questionnaire had a high level of internal consistency (Cronbach’s α = 0.80). Paired *t* tests showed no significant differences between two different occasions. Total and item scores differed significantly between patient and control groups. They concluded that the ESS is a simple and reliable method for measuring persistent daytime sleepiness and may be used in sleep researches in Turkish population.

### Quality of Life

The general health related quality of life (HRQL) instrument used in this study was the SF-36 Health Survey (SF-36), Version 1.0 (The Health Institute, New England Medical Center). The SF-36 measures the health domains of physical functioning, role limitations due to physical health problems, body pain, general health, vitality, social function, role limitations due to emotional problems, and mental health. These domains can be further aggregated into two summary scores: physical and mental health summary scores (Ware & Sherbourne, 1992).

Likert-type questions cover the last 4 weeks. For each scale scores range between 0–100. The lower the score, the worse the health status. The reliability and validity of the Turkish version of the SF-36 made by Koçyiğit, Aydemir, Fişek, Ölmez, and Memiş (1999).

Almost all of the domains of SF-36 discriminated ill and well persons and could be accepted as a very consistent profile sensitive to the functional status. Validity and reliability comparisons were made by testing convergent validity, content validity, discriminant validity and internal consistency. Pearson’s and Spearman’s correlation analysis, linear multiple regression analysis, Student’s *t* test and Cronbach alpha values were applied by using SPSS.

### Statistical Analysis

All clinical parameters were expressed as mean ± SD, as well as percentages (for categorical variables). The patients with OSA and the control group were compared using an unpaired *t* test for continuous parameters (Body Mass Index, Apnea-Hypopnea Index, Epworth Sleepiness Scale) and the Chi-squared test for categorical parameters (ADHD  % and sex %). Bivariate analyses were performed using Pearson correlation. A *p* value of <.05 was considered to be statistically significant.

## Results

Table 1 demonstrates the characteristics of patients with OSA and control group. The age and gender distribution in both groups (OSA and control) were similar. The quality of life in the two groups did not differ. As expected, Epworth Sleepiness Scale scores in OSA patients were higher than control group. The prevalence of adult attention deficit/hyperactivity disorder symptoms did not differ between the patients with OSA and the control group [(7.4 % (6/75) vs. 6.3 % (2/30), *p* = 0.8, respectively]. There were eight subjects with ADHD symptoms.

**Table 1:** The characteristics of patients with OSA and control group

	Control group (*n:*32)	Patients with OSA (*n:*81)	*p* value
Age, years	44.1 ± 13.2	48.1 ± 8.9	.1
Sex, M/F	20/12	66/15	.06
BMI	26.8 ± 4.4	29.8 ± 4.7	.003
ADHD %	6.3	7.4	.8
ESS	7.7 ± 5.3	10.6 ± 6.0	.03
SF36 physical component	68.9 ± 18.8	67.2 ± 22.8	.7
SF36 mental component	66.0 ± 20.8	64.1 ± 22.6	.6
Anxiety score	7.1 ± 4.5	7.1 ± 4.7	.9
Depression score	6.7 ± 3.9	7.0 ± 3.9	.7
Arousol index	19.1 ± 12.4	23.0 ± 15.4	.2
Basal O_2_	94.3 ± 1.3	92.6 ± 3.1	.005
Sleep duration	7.3 ± 1.6	7.6 ± 1.6	.4
Smoking	7.4 ± 13.4	14.7 ± 17.3	.0

*BMI* Body Mass Index, *ADHD* attention deficit hyperactivity disorder, *ESS* Epworth Sleepiness Scale

Table 2 demonstrates the characteristics of OSA patients without and with attention deficit/hyperactivity disorder symptoms.

**Table 2:** The characteristics of OSA patients without and with ADHD

	OSA patients with ADHD (*n:*6)	OSA patients without ADHD (*n:*75)	*p* Value
Age, years	46.8 ± 8.7	48.2 ± 9.0	.7
Sex, M/F	2/5	64/11	.009
BMI	32.4 ± 5.8	29.6 ± 4.6	.1
AHI	32.1 ± 11.5	49.1 ± 24.9	.1
ESS	17.0 ± 5.5	10.0 ± 5.8	.007
SF36 physical component	32.2 ± 14.3	70.1 ± 20.9	.0001
SF36 mental component	23.7 ± 30.1	67.4 ± 18.4	.0001
Anxiety score	14.0 ± 0.6	6.6 ± 4.4	.0001
Depression score	11.8 ± 1.8	6.8 ± 4.0	.002
Arousol index	12.6 ± 8.0	23.8 ± 15.5	.08
Basal O_2_	92.5 ± 2.0	92.6 ± 3.2	.9
Sleep duration	8.5 ± 3.2	7.5 ± 1.5	.1
Smoking	13.1 ± 15.7	14.8 ± 17.1	.8

*BMI* Body Mass Index, *AHI* Apnea-Hypopnea Index, *ESS* Epworth Sleepiness Scale

OSA patients with attention deficit/hyperactivity disorder symptoms, as compared with those without, had higher anxiety scores and poorer physical component scores of quality of life and higher Epworth Sleepiness Scale scores (Table 2). Accordingly, the presence of symptoms of ADHD appeared to negatively affect the quality of life, daytime sleepiness and psychological status of our sample.

ADHD scores in patients with OSA were associated with anxiety and depression scores and SF36 physical and mental component scores in bivariate analyses (Table 3).

**Table 3:** Correlates of ADHD scores in patients with OSA

	Anxiety score		Depression score		SF36 physical component		SF36 mental component		AHI		ESS	
	*r*	*p* value	*r*	*p* value	*r*	*p* value	*r*	*p* value	*r*	*p* value	*r*	*p* value
ADHD score	.65	.0001	.34	.002	−.40	.0001	−.55	.0001	.08	.4	.47	.0001

*ADHD* attention deficit hyperactivity disorder

## Discussion

The current investigation was conducted because it was hypothesized that an association between ADHD symptoms and OSA could be found in adults with OSA. Some studies have suggested an association between OSA and ADHD in children (Cohen-Zion & Ancoli-Israel, 2004; Huang et al., 2004; O’Brien et al., 2003).

However, little is known about this association in adults. Naseem et al. (2001) indicated the presence of OSA in three adults who were being treated for ADHD. Significant improvement in their daytime somnolence as well as psychosocial functioning was seen after therapy with nasal CPAP in two of these patients (Naseem et al., 2001). Nevertheless, this study is limited to three cases, the results cannot be generalized. Gau et al. (2007) indicated that ADHD symptoms in a community sample of young adults in Taiwan are related to sleep problems (dyssomnia, parasomnia, and snoring).

Polysomnography study of 5- to 7-year-old children by O’Brien et al. (2003) indicated that OSA was present in 5 % of those with significant ADHD symptoms, 26 % of those with mild symptoms, and 5 % of those with no symptoms. They concluded that sleep-disordered breathing (SDB) can lead to mild ADHD-like behaviors that can be readily misperceived and potentially delay the diagnosis and appropriate treatment. In a study by Huang et al. (2007) 55.2 % of children with ADHD had AHI > 1 events/h. They concluded that recognition and surgical treatment of underlying mild SDB in children with ADHD may prevent unnecessary long-term drug usage and the symptoms may improve. However, in another study in forty children aged 6–14 years with ADHD without observed apneic episodes in sleep or daytime sleepiness indicated that no patients had OSA on Polysomnography (Sangal, Owens, & Sangal, 2005).

All of the above studies have been conducted to evaluate the presence of sleep problems in ADHD patients. However, there is no study concerning the frequency of ADHD symptoms and its effects in OSA patients.

In our study, we found ADHD symptoms in 7.4 % of OSA patients. OSA patients with ADHD symptoms, as compared with those without, had higher anxiety scores and poorer physical component of quality of life scores and lower apnea-hypopnea index. ADHD scale scores were associated with anxiety and depression scores and physical and mental component quality of life in bivariate analyses. In actuality, depression, anxiety, sleep problems, memory problems, attentional and motivational problems, and quality of life problems are correlated with a significant number of serious psychological disorders and not just ADHD. Yet, studies concerning psychological functioning and quality of life in subjects with ADHD are scarce. In a study by Chao et al. (2008), the 328 young subjects in the ADHD group had more severe depressive, anxiety symptoms and daytime sleepiness, and had poorer quality of life than the 601 controls. The quality of life scores of seventy-two children aged 8–17 years with ADHD was lower than that of the control group matched for age and gender in another study (Jafari, Ghanizadeh, Akhondzadeh, & Mohammadi, 2011). As seen, the OSA with ADHD symptom may lead to further deterioration in daytime functions. The appropriate treatment of only OSA in these patients might not lead to sufficient improvement in daytime functions. Stimulant drugs are currently used in OSA patients for residual sleepiness. As known, ADHD is also treated with these drugs. The research by Looby and Earleywine (2011) indicated that placebo effects for prescription stimulants do influence subjective mood and may be implicated in nonmedical stimulant use. Thus, placebo effects should be considered in deciding the treatment of stimulant drugs.

As with all research this study has limitations that should be noted. Most notably scales have several limitations, and diagnoses should not be made on the bases of these data alone. To diagnose ADHD interviews with patients form the core of the clinical evaluation (Holperin, Newcorn, Schulz, & Sharma, 2006).

In addition, in OSA, a larger sample size might also have yielded a different finding about the overlap between OSA and ADHD. However, despite these limitations our findings showed that in our sample ADHD did not appear to be frequent in adult patients with OSA. But, the presence of ADHD in OSA did appear linked with poorer quality of life, the more anxiety and the excessive daytime sleepiness, presence of attention deficit/hyperactivity disorder symptom should be kept in mind.
